# The Cell Shape-determining Csd6 Protein from *Helicobacter pylori* Constitutes a New Family of l,d-Carboxypeptidase*

**DOI:** 10.1074/jbc.M115.658781

**Published:** 2015-08-25

**Authors:** Hyoun Sook Kim, Ha Na Im, Doo Ri An, Ji Young Yoon, Jun Young Jang, Shahriar Mobashery, Dusan Hesek, Mijoon Lee, Jakyung Yoo, Minghua Cui, Sun Choi, Cheolhee Kim, Nam Ki Lee, Soon-Jong Kim, Jin Young Kim, Geul Bang, Byung Woo Han, Byung Il Lee, Hye Jin Yoon, Se Won Suh

**Affiliations:** From the Departments of ‡ Chemistry and; ¶ Biophysics and Chemical Biology, College of Natural Sciences, and; § Research Institute of Pharmaceutical Sciences, College of Pharmacy, Seoul National University, Seoul 151-742, Republic of Korea,; the ‖ Department of Chemistry and Biochemistry, University of Notre Dame, Notre Dame, Indiana 46556,; the ** National Leading Research Laboratory of Molecular Modeling and Drug Design, College of Pharmacy, Graduate School of Pharmaceutical Sciences, and Global Top 5 Research Program, Ewha Womans University, Seoul 120-750, Republic of Korea,; the ‡‡ Department of Physics, POSTECH, Pohang 790-784, Republic of Korea,; the §§ Department of Chemistry, Mokpo National University, Chonnam 534-729, Republic of Korea,; the ¶¶ Division of Mass Spectrometry, Korea Basic Science Institute, Chungbuk 363-883, Republic of Korea, and; the ‖‖ Biomolecular Function Research Branch, Division of Convergence Technology, Research Institute, National Cancer Center, Gyeonggi 410-769, Republic of Korea

**Keywords:** cell motility, Helicobacter pylori, peptidoglycan, protein structure, structure-function, Csd6, HP0518, L,D-carboxypeptidase, cell shape, flagellin

## Abstract

*Helicobacter pylori* causes gastrointestinal diseases, including gastric cancer. Its high motility in the viscous gastric mucosa facilitates colonization of the human stomach and depends on the helical cell shape and the flagella. In *H. pylori*, Csd6 is one of the cell shape-determining proteins that play key roles in alteration of cross-linking or by trimming of peptidoglycan muropeptides. Csd6 is also involved in deglycosylation of the flagellar protein FlaA. To better understand its function, biochemical, biophysical, and structural characterizations were carried out. We show that Csd6 has a three-domain architecture and exists as a dimer in solution. The N-terminal domain plays a key role in dimerization. The middle catalytic domain resembles those of l,d-transpeptidases, but its pocket-shaped active site is uniquely defined by the four loops I to IV, among which loops I and III show the most distinct variations from the known l,d-transpeptidases. Mass analyses confirm that Csd6 functions only as an l,d-carboxypeptidase and not as an l,d-transpeptidase. The d-Ala-complexed structure suggests possible binding modes of both the substrate and product to the catalytic domain. The C-terminal nuclear transport factor 2-like domain possesses a deep pocket for possible binding of pseudaminic acid, and *in silico* docking supports its role in deglycosylation of flagellin. On the basis of these findings, it is proposed that *H. pylori* Csd6 and its homologs constitute a new family of l,d-carboxypeptidase. This work provides insights into the function of Csd6 in regulating the helical cell shape and motility of *H. pylori*.

## Teaser

**Background:** Csd6 is one of the cell shape-determining proteins in *H. pylori*.

**Results:** The active site of Csd6 is tailored to function as an l,d-carboxypeptidase in the peptidoglycan-trimming process.

**Conclusion:** Csd6 constitutes a new family of l,d-carboxypeptidase.

**Significance:** The substrate limitation of Csd6 is a strategy that *H. pylori* uses to regulate its helical cell shape and motility.

## Introduction

*Helicobacter pylori* is a spiral-shaped Gram-negative bacterium that colonizes the upper gastrointestinal tract in approximately half of the world's human population. Its infection of the gastric mucosa is associated with various gastric diseases, including chronic gastritis, peptic ulcer, mucosa-associated lymphoid tissue lymphoma, and gastric adenocarcinoma (1, 2). *H. pylori* is regarded as a primary factor for gastric cancer development (1) and was classified as a group I carcinogen by the International Agency for Research on Cancer of the World Health Organization. In recent years, *H. pylori* infection has also been implicated with some extra-digestive diseases (3). Typical treatment regimens for eradicating *H. pylori* consist of a proton pump inhibitor (for example, omeprazole) and the antibiotics such as clarithromycin and amoxicillin (or metronidazole). However, increasing drug resistance in *H. pylori* requires the discovery of new antibiotics (4).

High motility of *H. pylori* is important for its colonization of the human stomach and its survival in the viscous gastric mucosa (5–7). The helical cell shape of *H. pylori* is believed to facilitate penetration of the viscous epithelial mucous layer via a cork-screwing mechanism (8–10). Several *H. pylori* mutants with altered cell shapes exhibited attenuated colonization (11). The peptidoglycan layer, the major component of the bacterial cell wall, plays an essential role in withstanding the turgor pressure and in determining the cell shape (12, 13). It is made of linear polysaccharide chains that consist of alternating β-1,4-linked *N*-acetylglucosamine-*N*-acetylmuramic acid disaccharide units, with a pentapeptide linked to *N*-acetylmuramic acid (14). In *H. pylori*, the pentapeptide sequence is l-Ala^1^-γ-d-Glu^2^-*m*DAP^3^-d-Ala^4^-d-Ala^5^ (or -Gly^5^), where *m*DAP^5^ refers to *meso*-2,6-diaminopimelate, and the neighboring peptides are cross-linked exclusively by the 4→3 linkage between the main chain of d-Ala^4^ from one strand and the side chain of *m*DAP^3^ from another strand (15) to form a mesh-like structure termed peptidoglycan sacculus (16, 17). Colonization and infection by *H. pylori* also rely on the ability of the bacterium to move toward the part of gastric mucosa with more neutral pH. Besides the helical morphology, powerful flagella of *H. pylori* are responsible for its high motility through the viscous gastric mucous layer (5–7). The flagella provide a propulsive torque as well as a rotary movement of the cell body; a helical cell shape of *H. pylori* generates a corkscrew-like rotation (7–9).

In many bacteria, including *H. pylori*, the peptidoglycan layer is remodeled by a number of cell-wall hydrolases as well as synthetases for the peptidoglycan maturation, regulation of cell-wall growth, cell division, peptidoglycan turnover and recycling, cell lysis, and the release of peptidoglycan fragments for host-pathogen interactions (18). In *H. pylori*, an amidase AmiA (19), peptidases Csd1–4, potential regulators Csd5 and CcmA (20–22), and another peptidase Csd6 (23) are required to tailor the peptidoglycan layer to generate the helical cell shape. We have recently reported the crystal structures of both Csd4 (HP1075 in strain 26695) (24) and Csd3 (HP0506 in strain 26695) (25). Csd4 is a Zn^2+^-dependent d,l-carboxypeptidase (d,l-CPase) of the M14 metallopeptidase family and cleaves the γ-d-Glu^2^-*m*DAP^3^ bond of the muramyl tripeptide to produce the muramyl dipeptide (21, 24). Csd3 (also known as HdpA) belongs to the M23 metallopeptidase family and has both d,d-endopeptidase and d,d-carboxypeptidase (d,d-CPase) activities (22). Csd6 (encoded by the *hp0518* gene in *H. pylori* 26695 strain) was identified as another member of the peptidoglycan trimming pathway and also a cell-shape determinant of *H. pylori* (23). The transposon mutant *H. pylori* with the disruption of the *csd6* gene or the *csd6* deletion mutant displayed a straight rod shape and an increase in tetrapeptide-containing muropeptides (23). Incubation of the recombinant hexahistidine-tagged Csd6 with tetrapeptide-rich sacculi from the Δ*csd1csd6* mutant *H. pylori* resulted in complete conversion of the monomeric tetrapeptides to tripeptides (23). In *Campylobacter jejuni*, another helically shaped gastrointestinal pathogen, a homolog of Csd6 has been characterized as an l,d-CPase and was named as Pgp2 (26). Loss of *pgp2* resulted in the morphology defect, changes in the peptidoglycan muropeptide profile, reduced motility, and moreover, a decreased interaction with the host (26). Between Csd6 and Pgp2, an overall amino acid sequence identity of 36% and similarity of 58% exist. Given the significance of *m*DAP-containing muramyl tripeptide as an agonist for the cytosolic innate immune receptor Nod1 (27, 28), an increased amount of muramyl tripeptide as a result of l,d-CPase reaction by Pgp2 (or Csd6 from *H. pylori*) was suggested to effect the Nod1 activation and ultimately the NF-κB transcriptional activity (26). The complete absence of tripeptide-containing muropeptides in Δ*pgp2* peptidoglycan did indeed reflect the reduced Nod1 activation, despite no effect in either intracellular survival or IL-8 secretion (26).

A bioinformatics analysis on the basis of the amino acid sequence predicts that residues 67–200 of *H. pylori* Csd6 form a YkuD domain (formerly called ErfK/YbiS/YcfS/YnhG; “l,d-transpeptidase” catalytic domain; Pfam 03734) that can possibly catalyze nonclassical 3→3 cross-linking of peptidoglycan. The YkuD domain-containing l,d-transpeptidases (l,d-TPases) have been identified in a range of bacteria, including *Enterococcus faecium*, *Bacillus subtilis*, and *Mycobacterium tuberculosis* (7, 26, 29, 30). They generate 3→3 cross-linkages of peptidoglycan, instead of 4→3 cross-linking catalyzed by classical d,d-transpeptidases, resulting in high level resistance to β-lactam antibiotics (31) or in peptidoglycan remodeling for dormancy in *M. tuberculosis* (32). However, the *Helicobacter* peptidoglycan layer is cross-linked exclusively by 4→3 linkages (15, 20, 33). Consistent with the absence of 3→3 cross-linked muropeptides in the *H. pylori* peptidoglycan sacculus, Csd6 was shown to exhibit the l,d-CPase activity only with no “transpeptidase” cross-linking activity against the muropeptides (23). However, it still remains unanswered why Csd6 has no “transpeptidase” cross-linking activity, despite the presence of a putative “l,d-TPase” domain and conservation of the catalytic Cys/His residues.

In many bacterial pathogens, flagella are best known for conferring motility and virulence, as well as serving as an export apparatus for virulence factors (34) and sensing the viscosity of a medium (35). Flagellar filaments of *H. pylori* are composed of two copolymerized flagellins (FlaA and FlaB) (36), which are heavily *O*-glycosylated with pseudaminic acid (Pse5Ac7Ac; Pse), a sialic acid resembling sugar (37, 38). The regulation of such post-translational modification is critical for the assembly of functional flagella (37, 39). Interestingly, a previous work highlighted the unique role of *H. pylori* G27 Csd6 that involves deglycosylation of FlaA; the Δ*csd6* mutant exhibited altered motility to facilitate host-cell interaction and superior colonization (40). It suggests that Csd6 binds Pse molecules or deglycosylates *O*-glycosylated FlaA proteins.

To better understand the molecular function of *H. pylori* Csd6, its biochemical, biophysical, and structural characterizations have been performed in this study. Mass analyses using the synthetic muramyl peptides indicate that Csd6 functions only as the l,d-carboxypeptidase (l,d-CPase) and not as the l,d-transpeptidase (l,d-TPase). Analytical ultracentrifugation and single molecule fluorescence resonance energy transfer (FRET) analyses indicate that Csd6 exists as a dimer in solution. A Csd6 monomer has a three-domain architecture consisting of the N-terminal domain (NTD; Val-13–Asn-56), the middle l,d-CPase domain (Lys-67–Glu-202), and the C-terminal nuclear transport factor 2-like domain (NTF2-like domain; Thr-209–Lys-330). The NTD shows very remote structural similarity to other known protein structures and plays a key role in dimerization. The middle catalytic domain has an overall fold of the l,d-TPase domain, validating the bioinformatics prediction. However, here this catalytic domain is referred to as the “l,d-CPase” domain, because we as well as others show that Csd6 functions only as an l,d-CPase. The Csd6 l,d-CPase domain resembles those of well characterized l,d-TPases, but its pocket-shaped active site is uniquely defined by the four loops I–IV, among which loops I and III show the most distinct variations in sequence length and conformation from known l,d-TPases. The d-Ala-complexed structure suggests possible binding modes of both the substrate and product to the l,d-CPase domain. The NTF2-like domain possesses a deep pocket for possible binding of a hydrophobic ligand such as Pse, and an *in silico* docking study supports its role in the control of the glycosylation level of flagellin. This work provides further insights into the strategy of *H. pylori* for regulating its helical cell shape and motility, which are crucial for its virulence. The reported structural information would serve as the foundation in a search for new drug targets to fight infections by *H. pylori*.

## Experimental Procedures

### 

#### 

##### Protein Expression and Purification

The PCR-amplified *csd6* gene from *H. pylori* 26695 strain, encompassing residues Val-13–Lys-330 of the gene product (HP0518), was cloned into the expression vector pET-28b(+) (Novagen) to express the recombinant Csd6 protein fused with a His_6_-containing tag at its N terminus. It was overexpressed in *Escherichia coli* Rosetta2(DE3)pLysS cells using a Luria Broth culture medium. Protein expression was induced by 0.5 mm isopropyl β-d-thiogalactopyranoside, and the cells were incubated for an additional 20 h at 18 °C following growth to mid-log phase at 37 °C. The cells were lysed by sonication in buffer A (50 mm Tris-HCl, pH 7.9, 500 mm sodium chloride, and 50 mm imidazole) containing 10% (v/v) glycerol and 1 mm phenylmethylsulfonyl fluoride. The crude lysate was centrifuged at 36,000 × *g* for 1 h. The recombinant Csd6 protein showed a tendency to aggregate at 4 °C; therefore, it was purified at room temperature in two column chromatography steps. The supernatant was applied to an affinity chromatography column of HiTrap Chelating HP (GE Healthcare), which was previously equilibrated with buffer A. Upon eluting with a gradient of imidazole in the same buffer, the Csd6 protein was eluted at 120–150 mm imidazole concentration. The eluted protein was applied to a HiLoad XK-16 Superdex 200^TM^ column (GE Healthcare), which was previously equilibrated with 20 mm Tris-HCl, pH 7.9, and 150 mm sodium chloride. Fractions containing the Csd6 protein were pooled and concentrated to 5.8 mg ml^−1^ for crystallization using a YM10 ultrafiltration membrane (Amicon). This construct gave the best crystals in crystallization experiments. Other constructs were also tried for expression and crystallization. The Val-13–Lys-330 construct fused with a C-terminal His_6_-containing tag was not expressed in *E. coli* Rosetta2(DE3)pLysS cells. The constructs covering residues 4–330 or 17–330 were expressed at very low levels and were insoluble. The construct covering residues 1–330 (full-length) was expressed in a soluble form, but its crystals diffracted poorly to 7 Å only.

As a positive control in the l,d-TPase assay, Ldt_Mt2_, an l,d-TPase encoded by the *rv2518c* gene of *M. tuberculosis* H37Rv strain, was used. The N-terminal region (Leu-20–Ala-42) of Ldt_Mt2_ is predicted to form a putative transmembrane helix; the gene covering residues Ala-55–Ala-408, fused with a His_6_-containing tag at its C terminus, was cloned into pET-21a(+) (Novagen). The recombinant Ldt_Mt2_ was overexpressed and purified essentially as above.

##### Crystallization, X-ray Data Collection, and Structural Determination

To solve the phase problem by anomalous diffraction, ethyl mercury thiosalicylate (EMTS)-derivative crystals of the recombinant Csd6 were obtained at 23 °C by co-crystallization in the presence of EMTS by the sitting-drop vapor diffusion method. Sitting drops were prepared by mixing 3 μl of the protein solution and 2 μl of the reservoir solution (250 mm potassium nitrate, 18% (w/v) polyethylene glycol 3,350, and 10 mm EMTS). EMTS-derivatized crystals grew to approximate dimensions of 0.1 × 0.1 × 0.05 mm within a few days. Single-wavelength anomalous diffraction data were collected at 100 K from an EMTS-derivatized crystal of Csd6 using a cryoprotectant solution containing 25% (v/v) glycerol added to the reservoir solution (Table 1). The raw data were processed and scaled using the program suite HKL2000 (41). Two mercury sites in two monomers of Csd6 in the crystallographic asymmetric unit were located using the AutoSol program of the PHENIX software package (42). The initial phases were further improved by density modification using the automatic model building program Resolve (43). The initial model was improved through iterative cycles of model building with Coot (44) and refinement with Refmac5 of the CCP4 program suite (45, 46).

Native crystals of ligand-free Csd6 were grown at 23 °C by the sitting-drop vapor diffusion method. Sitting drops were prepared by mixing 0.5 μl of the protein solution, 0.1 μl of a 10-fold diluted microseed crystal solution, and 0.4 μl of the reservoir solution (1.5% (v/v) Tacsimate, pH 7.0, 22% (w/v) PEG 3,350, and 100 mm sodium-HEPES, pH 7.5). The microseed crystals were originally obtained with ligand-free Csd6 using the above reservoir solution. The crystals grew to approximate dimensions of 0.2 × 0.1 × 0.05 mm within a few days. X-ray diffraction data for the ligand-free Csd6 (Csd6-unbound) were collected at 100 K using a cryoprotectant solution containing 20% (v/v) glycerol added to the reservoir solution (Table 1). X-ray diffraction data for the d-Ala-bound Csd6 (Csd6-Ala) were collected at 100 K after the native crystal was preincubated for 2 min in a cryoprotectant solution containing both 20% (*v*/*v*) glycerol and 96 mm d-Ala, which were added to the reservoir solution (Table 1). The structures of both Csd6-unbound and Csd6-Ala were determined by molecular replacement with the program MolRep (47) using the refined model of EMTS-derivatized Csd6. Stereochemistry of the refined models was evaluated using MolProbity (48). Data collection and refinement statistics are given in Table 1.

##### In Silico Docking

Induced fit docking (IFD) (Schrödinger Suite Induced Fit Docking protocol; Glide version 5.7, Prime version 3.0, Schrödinger, LLC, New York) (49, 50) was employed to predict the binding mode of Pse with consideration of protein flexibility. The Csd6 structure (monomer A model) was prepared for docking calculations using the Protein Preparation Wizard implemented in Maestro (version 9.2, Schrödinger, LLC, New York). The structure of Pse was built in Maestro 9.2, and the possible conformations of the ligand were generated using LigPrep (version 2.5, Schrödinger, LLC, New York). Pse was docked onto Csd6 using the following steps. (i) The receptor grid of Csd6 was defined as an enclosing box at the centroid of the key amino acid residues (*i.e.* Trp-227, Lys-258, Tyr-297, Lys-313, and Glu-329) in the binding pocket. (ii) In the initial Glide docking stage, a potential docking with van der Waals radius scaling of 0.5 for the protein and ligand was performed retaining a maximum number of 20 poses per ligand. (iii) Residues within 5.0 Å of ligand poses were kept free to move in the Prime refinement step, and the side chains were further optimized. (iv) Poses within 30 kcal mol^−1^ of the energy cutoff in the previous step were re-docked using Glide XP. (v) The binding energy (IFDScore) for each output pose was computed as implemented in the IFD protocol. The best docked poses were also reproduced by a different docking program, Surflex-Dock of Sybyl X 2.0 (Tripos Int., St. Louis, MO). All the computations were undertaken on an Intel® Xeon^TM^ Quad-core 2.5 GHz workstation with Linux Cent OS release 5.5.

As a positive control of the docking method, IFD of sialic acid (*N*-acetylneuraminic acid) and Pse onto *Micromonospora viridifaciens* sialidase (PDB code 1EUS) (51) and *Pseudomonas aeruginosa* pseudaminidase (PDB code 2W38) (52) was performed. The docked conformation of sialic acid showed a root-mean-square (r.m.s.) deviation of 1.5 Å from the co-crystallized 2-deoxy-2,3-dideoxy-*N*-acetylneuraminic acid (a sialic acid mimic) in *M. viridifaciens* sialidase (51). The binding mode of Pse in *P. aeruginosa* pseudaminidase predicted by the present docking calculation was very similar to that of the reported docking result (52). These results validate the present docking method.

##### Peptidase Assay by Mass Analysis

**FIGURE 1.**
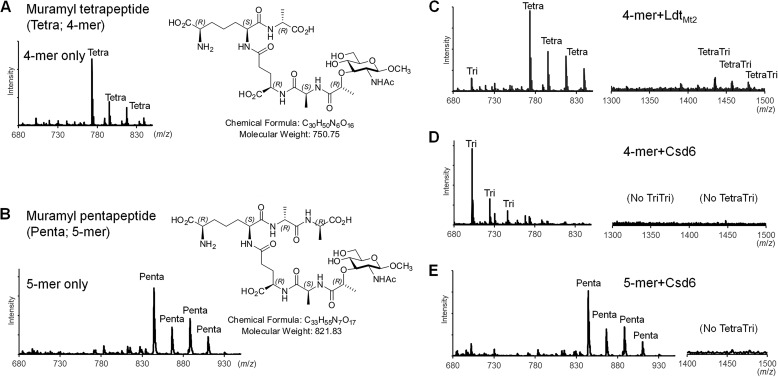
**l,d-CPase activity of Csd6.** *A* and *B*, mass spectra and structural formulas of the synthesized muramyl tetrapeptide (*A*) and muramyl pentapeptide (*B*). The observed peaks correspond to a series of sodium adduct ions of the peptides. *C–E*, mass spectra of the peptide samples upon incubation with Ldt_Mt2_ and Csd6. In the control reaction catalyzed by Ldt_Mt2_, the dimeric cross-linked tetra-tripeptide species is produced from the muramyl tetrapeptide (*C*). In contrast, in the reaction catalyzed by Csd6, the muramyl tetrapeptide is converted to the muramyl tripeptide but not to dimeric cross-linked tetra-tripeptide species (*D*). Upon incubation with Csd6, the muramyl pentapeptide undergoes no change (*E*). The observed *m*/*z* values for the charged species with bound sodium ions (up to four: [M + Na]^+1^, [M + 2Na-H]^+1^, [M + 3Na-2H]^+1^, and [M + 4Na-3H]^+1^) agree with the calculated *m*/*z* values for muramyl tri-, muramyl tetra-, muramyl penta-, or dimeric cross-linked tetra-tripeptides (average relative mass of neutral species = 679.67, 750.75, 821.83, or 1412.41, respectively).

As potential substrates, synthetic muramyl tetrapeptide (β-methyl *N*-acetylmuramic acid-l-Ala^1^-γ-d-Glu^2^-*m*DAP^3^-d-Ala^4^) and muramyl pentapeptide (β-methyl *N*-acetylmuramic acid-l-Ala^1^-γ-d-Glu^2^-*m*DAP^3^-d-Ala^4^-d-Ala^5^) were prepared as described previously (Fig. 1, *A* and *B*) (53). The analyte (muramyl tetrapeptide or muramyl pentapeptide) at 5 mm was incubated for 2 h at 37 °C with the recombinant Csd6 protein (5 μm), which was dissolved in 20 mm sodium phosphate, pH 6.0, and 150 mm sodium chloride. A sample solution (1 μl) was mixed on the target with a fresh saturated matrix solution of 2,5-dihydroxy benzoic acid dissolved in 0.1% (v/v) trifluoroacetic acid and 50% (v/v) acetonitrile. For sample deposition, a 384-position stainless steel sample plate was used. Mass spectra were acquired on a matrix-assisted laser desorption/ionization quadrupole ion trap time-of-flight mass spectrometer (MALDI-QIT-TOF MS, AXIMA QIT; Shimadzu/Kratos, Manchester, UK) equipped with a nitrogen laser (337 nm, 3-ns pulse width, maximum pulse rate of 10 Hz). Mass spectra were obtained in a positive ion mode. Helium was used for trapping and cooling ions in the ion source. The pressure in the trap was held at 4 × 10^−3^ torr. Each spectrum constituted an average of 200 profiles. All spectra were externally calibrated with bradykinin (757.3992 Da), angiotensin II (1046.5418 Da), angiotensin I (1296.6848 Da), Glu-fibrinopeptide B (1570.6768 Da), and *N*-acetyl renin substrate (1800.9432 Da) in TOFMix^TM^ (Shimadzu, Japan). Acquisition and data processing were controlled by the Lanchpad^TM^ software. The *m*/*z* values of multiple sodium adduct ions from the substrates and products are shown in Fig. 1, *A–E*. As a positive control, the peaks were detected corresponding to the dimeric cross-linked species between the muramyl tetrapeptide and muramyl tripeptide (Fig. 1*C*), a product of the l,d-TPase reaction catalyzed by the recombinant Ldt_Mt2_ (Ala-55–Ala-408). The l,d-TPase domain (residues Asp-251–Val-378) of *M. tuberculosis* Ldt_Mt2_ shows sequence identity and similarity of 22 and 38%, respectively, with the Csd6 l,d-CPase domain (residues Lys-67–Glu-202).

To evaluate the importance of specific residues in the l,d-CPase activity of Csd6, each of seven residues in the active site was mutated into alanine as follows: E110A, Y132A, Y133A, H155A, W158A, H160A, and C176A. The mutations were verified by DNA sequencing. The wild type and these single mutants of Csd6 were incubated with the substrate muramyl tetrapeptide for 30 min at 23 °C. Mass spectra were acquired as described above.

##### Analytical Ultracentrifugation

To determine the oligomeric state of the recombinant Csd6 in solution, equilibrium sedimentation and sedimentation velocity experiments were carried out using a Beckman ProteomeLab XL-A analytical ultracentrifuge in 20 mm Tris-HCl buffer, pH 7.9, containing 150 mm sodium chloride and 1.5 mm tris(2-carboxyethyl)phosphine at 20 °C. For the equilibrium sedimentation experiment, the absorbance from the Csd6 samples was measured at 235 and 280 nm using a six-sector cell at two speeds (16,000 and 20,000 rpm) and at three different Csd6 monomer concentrations (2.74, 3.65, and 4.56 μm) with a loading volume of 135 μl. For the sedimentation velocity experiment, the Csd6 samples at two different monomer concentrations (0.40 and 5.00 μm) were measured in double-sector cells at 30,000 rpm, and the collected data were analyzed by SEDFIT and SEDPHAT programs available on line. The concentration of the recombinant Csd6 protein was calculated using ϵ_280 nm_ = 54,780 m^−1^ cm^−1^.

##### Single Molecule Fluorescence Resonance Energy Transfer (FRET)

To analyze the dimeric form of Csd6 in solution, a single molecule FRET technique incorporating alternating laser excitation (ALEX) was employed (54, 55). Briefly, in this method the fluorescence signal of a dimeric molecule was analyzed one-by-one, and the result is presented in a two-dimensional *E-S* graph (Fig. 3*A*), where *E* is the FRET efficiency and *S* denotes the Cy3/Cy5 molar ratio in a dimer. The Csd6 sample was divided into 2 aliquots, each of which was labeled on the sole cysteine residue (Cys-176) with the maleimide-reactive Cy3 dye (donor) or the Cy5 dye (acceptor). Subsequently, Cy3-labeled Csd6 (Cy3-Csd6) and Cy5-labeled Csd6 (Cy5-Csd6) were mixed at 2 μm final monomer concentration in a buffer (20 mm Tris-HCl, pH 7.9, and 150 mm sodium chloride) for 3 h at 37 °C. The protein sample was diluted further to 100 pm using a single molecule buffer (20 mm Tris-HCl, pH 7.9, 150 mm sodium chloride, 5% (v/v) glycerol, 1 mm 2-mercaptoethylamine, and 0.01% (v/v) bovine serum albumin). The single molecule data were acquired from a 10-min measurement.

##### Surface Plasmon Resonance Experiment

The kinetics and affinity of Csd6 with the synthetic muramyl tetrapeptide and muramyl tripeptide (β-methyl *N*-acetylmuramic acid-l-Ala^1^-γ-d-Glu^2^-*m*DAP^3^) as a reaction product were assessed using a Reichert SR7500 surface plasmon resonance (SPR) dual channel instrument (Reichert, Depew, NY). Purified Csd6 in 20 mm sodium acetate, pH 5.5, was immobilized using the standard amino coupling at 20 μl min^−1^ on a carboxymethyl dextran hydrogel surface sensor chip (Reichert, Depew, NY) until saturation was achieved. The running buffer B used in all SPR experiments was 20 mm HEPES, pH 7.9, and 150 mm sodium chloride. SPR experiments were performed at 25 °C. The muramyl tetrapeptide or muramyl tripeptide at concentrations of 15.6, 31.3, 62.5, 125, 250, and 500 μm was injected over the Csd6-chip at 30 μl min^−1^ for 5 min for association analyses. Subsequently, the running buffer was flowed over the chip for an additional 6 min (30 μl min^−1^) for dissociation analyses. Regeneration of the chip was carried out using 20 mm sodium hydroxide. Binding was detected as a change in the refractive index at the surface of the chip as measured by the response unit. A reference flow cell was used to record the response by bovine serum albumin (BSA) as a positive control, and the response by BSA was subtracted from each sample. SPR data were fit using the Scrubber2 software.

## Results

### 

#### 

##### Csd6 Functions as an l,d-CPase but Not as an l,d-TPase

To verify the molecular function of *H. pylori* Csd6 in peptidoglycan modification, the peptidase activity of Csd6 was measured *in vitro* with the synthetic muramyl tetrapeptide and muramyl pentapeptide (Fig. 1, *A* and *B*) as potential substrates using mass analyses. When the muramyl pentapeptide was incubated with the recombinant Csd6(13–330), no reaction product could be detected (Fig. 1*C*). This suggests that Csd6 does not have “endo”-, “d,d-carboxy”-, or “l,d-trans”-peptidase activities with the muramyl pentapeptide. The l,d-CPase domain of Csd6 shows limited but significant sequence identity (22%) with the l,d-TPase domain of *M. tuberculosis* Ldt_Mt2_. *M. tuberculosis* Ldt_Mt2_ is a functional l,d-TPase, catalyzing the formation of 3→3 cross-links between the muramyl tetrapeptide and the muramyl tripeptide (56). Therefore, as a positive control for measuring the l,d-TPase activity, the muramyl tetrapeptide was incubated with the recombinant *M. tuberculosis* Ldt_Mt2_. In this control reaction, not only the muramyl tripeptide (*Tri* in Fig. 1*C*) but also the dimeric cross-linked species (*Tetra-Tri* in Fig. 1*C*) were detected as the reaction products. In comparison, when the muramyl tetrapeptide was incubated with the recombinant Csd6, the muramyl tripeptide was detected as the sole reaction product of trimming without any dimeric cross-linked species (Fig. 1*D*). This result implies that Csd6 would not be active for cross-linking, if the muramyl tripeptide were used as a substrate. The present data, together with the previous report that Csd6 converts monomeric tetrapeptides of the peptidoglycan sacculus into tripeptides (23), clearly establish that *H. pylori* Csd6 functions as an l,d-“carboxypeptidase” devoid of a cross-linking l,d-TPase, despite the presence of the conserved Cys/His residues, which are characteristics of the l,d-TPase domains. The active-site features of Csd6, as discussed in more detail below, explain why Csd6 is inactive as l,d-TPase.

##### Csd6 Monomer Is Organized into a Three-domain Architecture

**FIGURE 2.**
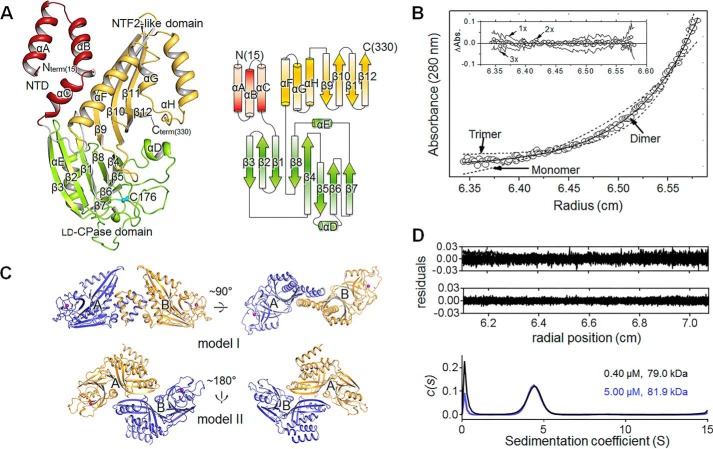
**Overall structure and the oligomeric state of *H. pylori* Csd6.** *A*, ribbon diagram (*upper panel*) and topology diagram (*bottom panel*) of the Csd6-unbound structure. The NTD, l,d-CPase domain, and NTF2-like domain are shown in *red, green,* and *yellow*, respectively. The secondary structure elements have been defined by the DSSP program (77). The nucleophile Cys-176 of the l,d-CPase domain is shown in a *cyan stick* model. *B*, equilibrium sedimentation data for Csd6 at an ultracentrifugal speed of 16,000 rpm using 3.65 μm protein at 20 °C. The *circles* are experimental data, and the *solid line* is a fitting line for an ideal monomer model. The *two dotted lines* are fitting lines for ideal monomer and trimer models. Distributions of the residuals for monomer, dimer, and trimer models are shown in the *inset panel*. These data indicate that Csd6 exists as homogeneous dimers in solution. *C*, two putative models of the Csd6 dimer in the crystal. Dimer model I (*upper panel*) and model II (*bottom panel*) are shown in ribbon diagrams. The side chain sulfur atoms of Cys-176 are shown as *purple spheres*. Model I is favored by the single molecule ALEX-FRET data (shown in Fig. 3*A*). *D*, plots of the residuals for dimer species model of Csd6 at 0.40 μm (*upper*) and 5.00 μm (*lower*), and the distribution of sedimentation coefficient (*c*(s) *versus s*, where *s* is in Svedberg unit, S) from the sedimentation velocity experiments.

To provide a structural basis of understanding the molecular function, the crystal structures of Csd6 have been determined, both in the ligand-free state (“Csd6-unbound”) (Fig. 2*A*) and in the d-Ala-bound state (“Csd6-Ala”) (Table 1). Refined models of both structures are essentially identical to each other with an r.m.s. deviation of 0.17 Å for 632 eq Cα positions. They account for residues Met-15–Lys-330 in each of two Csd6 monomers in an asymmetric unit (ASU). In the case of Csd6-unbound structure, two monomers in the ASU were similar to each other with an r.m.s. deviation of 0.64 Å for 316 Cα atom pairs, except for the N-terminal residues (Leu-15–Asp-41) showing r.m.s. deviations greater than 1.0 Å with a maximum Cα deviation (7.6 Å) at Leu-15. For the Csd6-Ala model, two monomers in the ASU agree with an r.m.s. deviation of 0.61 Å for 316 Cα atom pairs, except for the N-terminal residues (Leu-15–Asp-41) showing r.m.s. deviations greater than 1.0 Å with a maximum Cα deviation (8.1 Å) at Leu-15. The amino acid residues with the largest deviation encompass the entire helix αA and the N-terminal half of αB.

**TABLE 1:** **Data collection and refinement statistics**

**A. Data collection**			
Data set	EMTS peak	Csd6-unbound	Csd6-Ala
Beamline source*^a^*	PLS BL-4A	PF BL-1A	PLS BL-5C
Space group	*P*2_1_2_1_2_1_	*P*2_1_2_1_2_1_	*P*2_1_2_1_2_1_
*a, b, c* (Å)	61.8, 89.6, 128.7	63.0, 91.0, 127.8	63.4, 90.6, 127.9
X-ray wavelength (Å)	1.0048	1.0000	0.9796
Resolution range*^b^* (Å)	50.0–2.90 (2.95–2.90)	50.0–2.03 (2.07–2.03)	50.0–2.04 (2.08–2.04)
No. of total reflections*^b^*	373,689 (18,354)	229,193 (11,715)	227,623 (11,074)
No. of unique reflections*^b^*	30,311 (1,515)*^c^*	47,555 (2,343)	47,575 (2,307)
Completeness (%)*^b^*	99.9 (100)*^c^*	98.3 (98.1)	99.9 (100)
〈*I*〉/〈σ*_I_*〉*^b^*	35.0 (4.7)*^c^*	25.0 (3.0)	29.3 (3.4)
Wilson *B* factor (Å^2^)	53.1	34.2	30.5
*R*_merge_*^b,d^* (%)	11.0 (73.1)*^c^*	9.5 (47.0)	8.6 (53.7)

**B. SAD phasing**	Figure of merit (before/after density modification) 0.34/0.66		

**C. Model refinement**			
PDB ID code		4XZZ	4Y4V
Resolution range (Å)		30.0–2.03	20.0–2.04
*R*_work_/*R*_free_*^e^* (%)		19.6/24.8	19.1/24.2
No. of non-hydrogen atoms/average *B*-factor (Å^2^)			
Total		5,589/40.6	5,606/37.8
Protein		5,248/40.4	5,249/38.7
Water oxygen		323/41.5	315/40.5
Glycerol		18/63.4	24/58.9
d-Ala		−/−	18/44.6
R.m.s. deviations from ideal geometry			
Bond lengths (Å)/angles (°)		0.009/1.33	0.010/1.39
R.m.s. *Z*-scores			
Bond lengths (Å)/angles (°)		0.46/0.61	0.49/0.65
Ramachandran (%)*^f^*			
Favored/outliers		98.6/0.00	98.4/0.00
Poor rotamers (%)*^f^*		0.00	0.70

*^a^* PF and PLS stand for Photon Factory, Japan, and Pohang Light Source, Korea, respectively.

*^b^* Values in parentheses refer to the highest resolution shell.

*^c^* Friedel pairs were treated as separate observations.

*^d^ R*_merge_ = Σ*_h_*Σ*_i_*|*I*(*h*)*_i_* − 〈*I*(*h*)〉|/Σ*_h_*Σ*_i_I*(*h*)*_i_*, where *I*(*h*) is the intensity of reflection *h*; Σ*_h_* is the sum over all reflections, and Σ*_i_* is the sum over *i* measurements of reflection *h*.

*^e^ R*_work_ = Σ‖*F*_obs_| − |*F*_calc_‖/Σ|*F*_obs_|, where *R*_free_ is calculated for a randomly chosen 5% of reflections, which were not used for structure refinement, and *R*_work_ is calculated for the remaining reflections.

*^f^* Values were obtained using MolProbity.

The Csd6 monomer consists of three domains of unequal sizes as follows: the NTD (Val-13–Asn-56), the middle l,d-CPase domain (Lys-67–Glu-202), and the C-terminal NTF2-like domain (Thr-209–Lys-330) (Fig. 2*A*). The NTD consists of three α-helices (αA–αC), and the helix αC is positioned at one end of the antiparallel coiled-coil formed by helices αA and αB. The l,d-CPase domain consists of two curved β-sheets (β3↓-β2↑-β1↓-β8↓-β4↑ and β4↑-β5↓-β6↑-β7↑), which share the β4-strand, as well as two α-helices (αD and αE). The NTF2-like domain forms an antiparallel four-stranded β-sheet (β9↓-β10↑-β11↓-β12↑), whose concave side is packed by three α-helices (αF–αH). DALI structural similarity searches (57) revealed no significant match with either the entire Csd6 with three domains or any combination of two connected domains in Csd6, suggesting that the three-domain architecture of Csd6 is unique. This is in line with our observation that the organization of the l,d-CPase domain and the NTF2-like domain in Csd6 and its homologs is unique among the l,d-TPase and l,d-CPase families according to the conserved domain searches using the InterPro database (58).

In the Csd6 monomer structure, three domains are arranged in a tripartite leaf-like fashion with a single chain connecting two adjacent domains (Fig. 2*A*). Virtually no interaction exists between the NTD and the l,d-CPase domain; the buried surface area at the interface is only 36 Å^2^, as calculated by the PISA server (59). Much more extensive interactions exist between the l,d-CPase domain and the NTF2-like domain, with a buried surface area of 718 Å^2^. The latter interface is mainly formed between strands β4 and β8 of the l,d-CPase domain and strands β9–β11 of the NTF2-like domain. It is primarily lined with hydrophobic residues of both domains (Val-119, Tyr-120, Tyr-146, Phe-197, and Ile-199 of the l,d-CPase domain and Ile-278, Tyr-280, Tyr-290, Val-292, and Tyr-316 of the NTF2-like domain). The NTD (helix αC) and the NTF2-like domain (helix αF) interact with each other, burying a surface area of 410 Å^2^ at the interface.

##### NTD of Csd6 Plays a Dominant Role in Homodimerization

**FIGURE 3.**
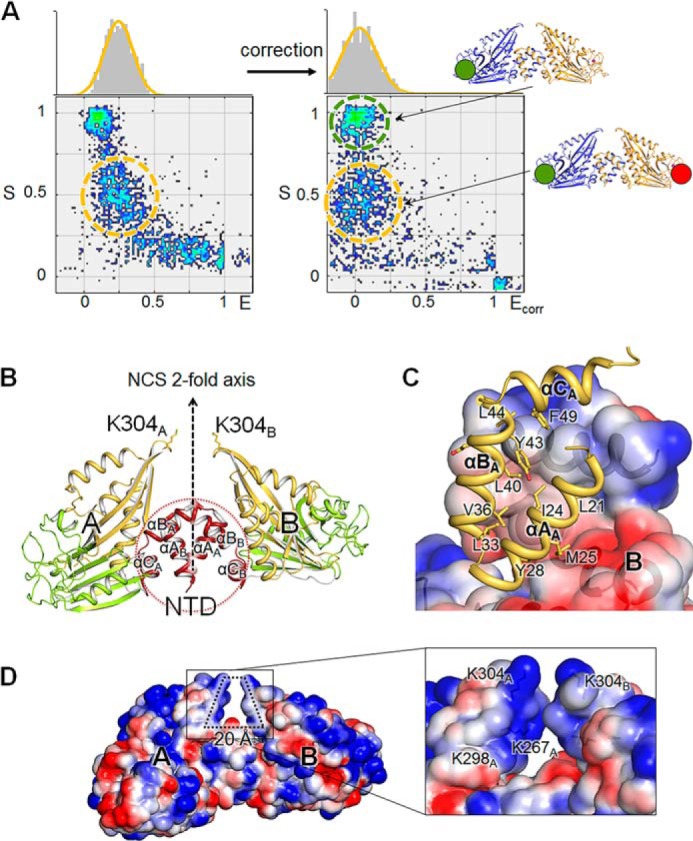
**Dimeric structure of *H. pylori* Csd6.** *A*, determination of the dimeric model of Csd6 by single molecule FRET technique. Single molecule FRET data are presented in a two-dimensional *E-S* graph, where *E* is the FRET efficiency and *S* denotes the Cy3 (donor)/Cy5 (acceptor) molar ratio in a dimer (54). Three clusters correspond to the dimers formed as follows: (i) between two Cy3-Csd6 monomers; (ii) between two Cy5-Csd6 monomers; and (iii) between a Cy3-Csd6 monomer and a Cy5-Csd6 monomer. The cluster appearing at S ∼ 1 (*green dotted ellipse*) with *E* ≈ 0 corresponds to Cy3-labeled Csd6 dimers. Csd6 dimers labeled with both Cy3 and Cy5 appear at S ∼ 0.5 (*orange dotted ellipse*) (55). *B*, ribbon diagram of the Csd6 homodimer (model I) colored as in Fig. 2*A*. In this dimer, A and B monomers are related by a noncrystallographic pseudo 2-fold symmetry. *C*, hydrophobic interactions between the NTDs of A and B monomers, which are shown in the ribbon diagram and the electrostatic surface diagram, respectively. *D*, electrostatic potential surface diagrams of the Csd6 dimer molecule. A detailed view of the specific hole formed by the dimerization is shown in the *black lined box*.

Equilibrium sedimentation measurements indicate that the Csd6 protein exists as a homogeneous dimer in solution (at the tested Csd6 monomer concentration range of 2.74–4.56 μm). The data fit well to a dimer model, and a representative result measured at 16,000 rpm using the 3.65 μm monomer concentration is presented in Fig. 2*B*. Further sedimentation velocity experiments at 0.40 and 5.00 μm Csd6 monomer concentrations also indicate the oligomeric state of Csd6 as a dimer, without any discernable dissociation of a dimer into monomers, down to 0.40 μm (Fig. 2*D*). In the crystal of Csd6, two possible models of the dimer could be identified. The dimer model I has a buried surface area of 1,080 Å^2^ per monomer (6.7% of the monomer surface area) at the interface, as analyzed by the PISA server (Fig. 2*C*, *upper panel*) (59). In the dimer model I, the two monomers are related by noncrystallographic “pseudo” 2-fold symmetry. The 2-fold symmetry is broken primarily by the significant difference in the orientation of N-terminal helices αA and αB between the two monomers. The dimer model II has a buried surface area of 726 Å^2^ (4.5% of the monomer surface area) (Fig. 2*C*, *lower panel*). In the dimer model II, the two monomers are related by an exact crystallographic 2-fold symmetry. The Δ^i^*G* values (solvation free energy gain upon formation of the interface) are −15.8 and −2.1 kcal mol^−1^ for models I and II, with the complexation significance scores of 1.000 and 0.000, respectively. These complexation significance score values imply that the interface in model I, but not in model II, plays an essential role in complex formation. Therefore, we conclude that the dimer model I in the crystal represents the biologically relevant Csd6 dimers in solution. This conclusion is supported experimentally by the distance between two Cys-176 residues in the dimer as estimated using the single molecule ALEX-FRET technique (Fig. 3*A*). The single molecule ALEX-FRET experiment was performed at 100 pm monomer concentration, which is much lower than that used for analytical ultracentrifugation, to avoid ensemble averaging (*i.e.* to avoid the case of having two noninteracting species) (60). After direct excitation leakage and buffer background corrections, the distance between Cy3 and Cy5 dipoles in the dye-labeled Csd6 dimer is estimated to be 107 Å from the corrected FRET efficiency (*E*_corr_ ≈0.03), using *R_o_* (Förster distance) of 60 Å for the Cy3 and Cy5 pair. In the crystal structure of Csd6-unbound, the distance between sulfur atoms of two Cys-176 residues is 96 and 66 Å in dimer models I and II, respectively. This result unambiguously favors model I, supporting the assignment of dimer model I in the crystal as the dimer in solution (Fig. 2*C*, *upper panel*).

The Csd6 dimer (*i.e.* dimer model I) is elongated with approximate dimensions of 100 × 80 × 50 Å (Fig. 3*B*). In this dimer, two l,d-CPase domains are well separated from each other, and their active sites are open toward the bulk solvent (Fig. 3*B*). The bulk of the buried surface area at the dimerization interface is contributed by the NTD (82.7%), with the linker between the NTD and the l,d-CPase domain contributing 5.7% and the NTF2-like domain contributing 11.6%. The N-terminal helix αA of the NTD takes slightly different conformations in the two monomers, with a maximum Cα deviation of 8.1 Å at Leu-15. The two NTDs pack against each other through their antiparallel coiled-coils (helices αA and αB), at an angle of about 60°, to form a four-helix bundle (Fig. 3*B*); numerous hydrophobic side chains are present at the interface. The αC-helices from two NTDs cover the sides of the four-helix bundle. Seven hydrogen bonds (involving Arg-26, Tyr-28, Gln-29, Gly-37, Asp-41, Glu-53, Tyr-63, Gln-65, and Phe-272) and a salt bridge (involving Arg-26 and Glu-53) exist at the interface (Fig. 3*C*). Interestingly, the Csd6 dimer has a deep crevice of an approximate size of 20 × 20 Å, with a widened bottom (indicated by *dotted lines* in Fig. 3*D*). The NTDs provide the bottom of the crevice, and the C-terminal NTF2-like domains form the two sides of the crevice. The sides of the crevice are lined with positively charged residues (Lys-267, Lys-298, and Lys-304) but no negatively charged residues (Fig. 3*D*).

A DALI search with the Csd6 NTD (Val-13–Asn-56) alone showed very remote structural similarities to other known protein structures. The highest *Z*-score was obtained with human phosphatidylinositol-4,5-bisphosphate 3-kinase catalytic subunit α isoform (PDB code 4L2Y-A; an r.m.s. deviation of 2.1 Å for 41 equivalent Cα positions in residues 16–56, a *Z*-score of 4.6, and a sequence identity of 5%). The Csd6 NTD is perhaps functionally more related to the L27N domain of *Mus musculus* PALS1-associated tight junction protein (PDB code 1VF6-D; an r.m.s. deviation of 4.2 Å for 41 eq Cα positions in residues 16-56, a *Z*-score of 4.3, and a sequence identity of 12%) (61). L27 domains, such as PALS1-L27N, have been established as a protein-binding module that brings multiple proteins into complexes for signaling, cell polarity, and epithelial morphogenesis (62, 63). Like the Csd6 NTD, the L27 domain is composed of three α-helices. However, unlike the Csd6 NTD that plays a key role in homodimerization, two different L27 domains (*e.g.* PALS1-L27N domain and PATJ-L27) heterodimerize by building a compact four-helix bundle structure through the first two helices from each L27 domain (61).

##### Active-site of Csd6 l,d-CPase Domain Is Tailored for the l,d-CPase Activity

**FIGURE 4.**
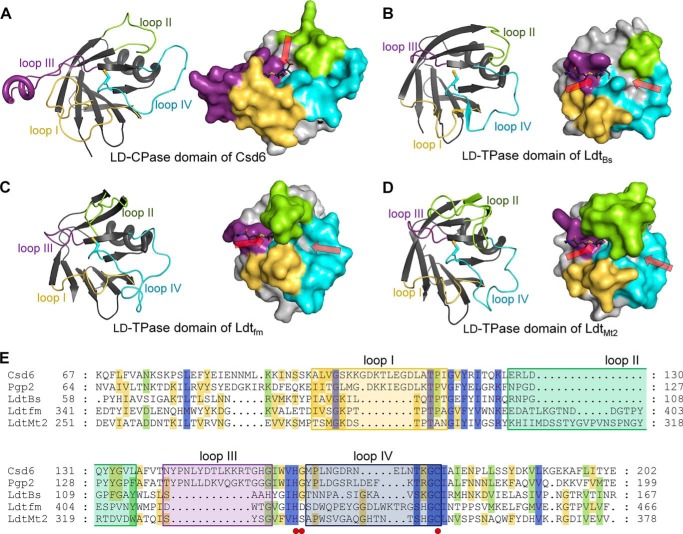
**Comparison of Csd6 and three l,d-TPases.** *A–D*, the ribbon diagrams (*left panels*) and surface diagrams (*right panels*) of the l,d-CPase domain of *H. pylori* Csd6 (*A*) and the l,d-TPase domains of *B. subtilis* Ldt_Bs_ (*B*), *E. faecium* Ldt_fm_ (*C*), and *M. tuberculosis* Ldt_Mt2_ (*D*). The four loops I–IV are colored in *yellow, green, purple*, and *light blue*, respectively. One or two paths that allow access to the catalytic triad are indicated by *red arrows*. In the meropenem-complexed Ldt_Mt2_ structure (PDB code 4GSU), the paths A and B correspond to *left* and *right arrows*, respectively (30). Meropenem was modeled into each domain structure by structural superimposition of the corresponding domains using the meropenem-complexed Ldt_Mt2_ (PDB code 4GSU). *E*, structure-based sequence alignment was performed by PROMALS3D (80), and the alignment was presented by GeneDoc. Structures of the l,d-CPase domain of *H. pylori* Csd6 (Swiss-Prot accession code O25255; PDB code 4XZZ) and l,d-TPase catalytic domains of *B. subtilis* Ldt_Bs_ (O34816; 1Y7M), *E. faecium* Ldt_fm_ (Q3Y185; 1ZAT), and *M. tuberculosis* Ldt_Mt2_ (O53223; 4GSU) were used in the alignment. In the case of *C. jejuni* Pgp2 (A1VZP0), no structural information was available, and thus only the sequence is used. *Red dots* indicate the catalytic triad (His-160, Gly-161, and Cys-176 in *H. pylori* Csd6). The four loops I–IV are shown in *colored boxes* as in *A*.

A bioinformatics analysis on the basis of amino acid sequence predicts *H. pylori* Csd6 to have an overall fold of the l,d-TPase catalytic domain over residues 67–200. The structure reported here validates this prediction (Figs. 2*A* and 4*A*). However, this catalytic domain is referred to as the l,d-CPase domain, because Csd6 is shown to function only as an l,d-CPase and not as an l,d-TPase against the muramyl tetrapeptide. According to the DALI search, the overall fold of the Csd6 l,d-CPase domain (Lys-67–Glu-202) (Fig. 4*A*) resembles those of well characterized l,d-TPases such as *B. subtilis* Ldt_Bs_ (Fig. 4*B*) (PDB code 1Y7M; a *Z*-score of 14.8, an r.m.s. deviation of 1.8 Å, and a sequence identity of 13% for 109 equivalent Cα positions) (64), *E. faecium* Ldt_fm_ (Fig. 4*C*) (PDB code 1ZAT; a *Z*-score of 12.1, an r.m.s. deviation of 2.7 Å, and a sequence identity of 14% for 111 eq Cα positions) (29), and *M. tuberculosis* Ldt_Mt2_ (Fig. 4*D*) (PDB code 4GSU; a *Z*-score of 11.7, an r.m.s. deviation of 2.1 Å, and a sequence identity of 14% for 107 eq Cα positions) (30). Unlike Csd6, all of these l,d-TPases are monomeric enzymes (29, 30, 64). Furthermore, the l,d-CPase domain of Csd6 contains the characteristic sequence motif **H**(S/G/D)*X*_14–19_**C** (Fig. 4*E*), where *X* stands for any amino acid and the strictly conserved residues are in boldface. This motif is conserved among proteins containing an l,d-TPase catalytic domain and provides the catalytic triad residues, which form a hydrogen bond network in a wide range of Cys/Ser-based proteolytic enzymes (65). The catalytic triad of Csd6 consists of His-160, Gly-161, and Cys-176 (marked by *red dots* in Fig. 4*E*), corresponding to His-336, Ser-337, and Cys-354 of Ldt_Mt2_ (30). However, *H. pylori* Csd6 is unrelated in its sequence and structure to previously characterized l,d-CPases such as l,d-carboxypeptidases A (LdcA) from *P. aeruginosa* (66) and *Novosphingobium aromaticivorans* DSM 12444 (67), and l,d-carboxypeptidases B (LdcB) from *Streptococcus pneumoniae*, *Bacillus anthracis*, and *Bacillus subtilis* (68). LdcA is a Ser-based l,d-CPase. The latter three LdcB proteins belong to the LAS (lysostaphin, d-Ala-d-Ala metallopeptidases, sonic hedgehog) family of Zn^2+^-dependent peptidases (68). This raises an intriguing question why *H. pylori* Csd6 functions only as an l,d-CPase, and not as an l,d-TPase, despite the overall l,d-TPase fold and the presence of the conserved catalytic triad.

A structural comparison of Csd6 l,d-CPase domain with l,d-TPase domains reveals that the shape of the active site in the Csd6 l,d-CPase domain seems to be tailored for the l,d-carboxypeptidation, *i.e.* the active site is a deep pocket and the catalytic triad positioned at the bottom of this pocket is accessible via a single narrow path (Fig. 4*A*), as in typical carboxypeptidases (69). In contrast, the active site in the corresponding domains of the above three l,d-TPases is an elongated groove, and the catalytic triad is accessible via two paths (Fig. 4, *B–D*) (30). The different shapes of the active sites are primarily dictated by the length and conformation of the loops around the entrance(s) to the active sites. Structural comparison and sequence alignment of the Csd6 l,d-CPase domain with the corresponding domains of three l,d-TPases (Fig. 4) show that unique features of Csd6 that distinguish it from other l,d-TPases reside primarily in the following four loop regions: loop I (β3-β4 loop; Ala-98–Ile-117); loop II (β4-β5 loop; Leu-126–Leu-136); loop III (β5-αD-β6 loop; Asn-141–Ile-157); and loop IV (β6-β7 loop; Met-162–Cys-176).

The reaction catalyzed by l,d-TPases such as Ldt_Mt2_ occurs in two sequential steps as follows: acylation of the donor substrate (“carboxypeptidation”) and deacylation by the acceptor substrate for cross-linking (“transpeptidation”) (70). As a consequence, l,d-TPases such as Ldt_Mt2_ have two accessible paths for donor and acceptor substrates (indicated by *red arrows* in Fig. 4, *B–D*), thereby allowing an approach of the acceptor substrate upon acylation of the donor substrate by the catalytic Cys residue for transpeptidation. For a better understanding of the substrate-binding site (Fig. 4, *A–D*), meropenem (mimicking the substrate) was modeled into Csd6 by superimposing its l,d-CPase domain and the l,d-TPase domain of the meropenem-complexed Ldt_Mt2_ (30). The position of the covalently bound meropenem represents the path for the donor substrate (“path A”). The active-site clefts in three l,d-TPases reveal a long groove for binding two substrates, which is contributed by the four loops I–IV (Fig. 4, *B–D*). In Ldt_Mt2_ and Ldt_fm_, the extended loop II (*green* colored in Fig. 4, *C* and *D*) forms a lid to cover the active-site cleft, while exposing two discrete paths for the access of two substrates. However, the loop II of Ldt_Bs_ is much shorter, and the active-site cleft is not covered with a lid (*green* colored in Fig. 4*B*). Like Ldt_Bs_, the loop II is much shorter in Csd6, and the active-site cleft is not covered with a lid. Moreover, in the Csd6 structure, the corresponding paths to the active-site cleft for access of two substrates in three l,d-TPases are blocked due to the distinct length and conformations of the loops I–IV, and the active-site cleft opens vertically toward the solvent (indicated by a *red arrow* in Fig. 4*A*), allowing access of only one muramyl peptide for carboxypeptidation. This difference in the shape of the active-site cleft is the likely reason for the enzyme serving as a carboxypeptidase, to the exclusion of the transpeptidase activity.

Among the four loops I–IV, the loops I and III show the most distinct variations in sequence length and conformation. They are considerably longer (8 and 12 residues, respectively) in *H. pylori* Csd6 than in the l,d-TPases (Fig. 4*E*). Loop III contains the helix αD, which protrudes from the active site. Together with loop I, it provides one side of the active-site cleft to recognize a substrate. The *C. jejuni* Pgp2, a Csd6 homolog, is predicted to contain an l,d-TPase domain on the basis of its amino acid sequence (Fig. 4*E*), but it was shown to function as an l,d-CPase (26). We notice that the four loops of Pgp2 show a highly similar pattern with those of Csd6 (Fig. 4*E*). Therefore, it is anticipated that the observed unique structural features of these four loops around the active-site cleft are conserved among Csd6 homologs in other ϵ-proteobacteria.

##### d-Ala-complexed Structure Reveals Binding Modes of Both the Substrate and Product

**FIGURE 5.**
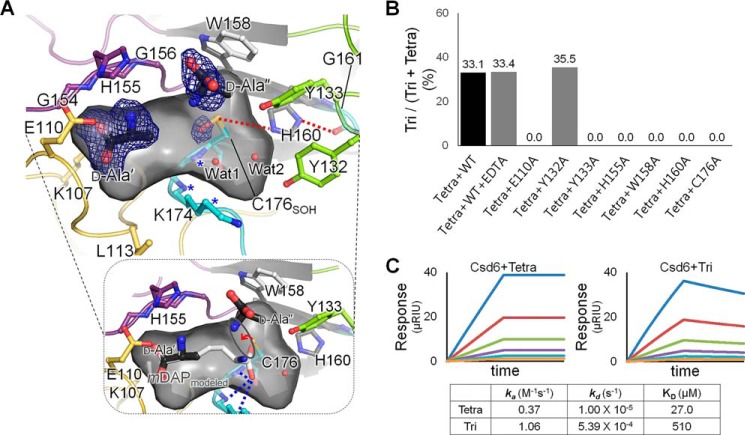
**d-Ala-complexed Csd6 and key residues for the l,d-CPase activity.** *A*, ribbon diagram and the accessible inner surface of the active site in the Csd4-Ala structure. The bound d-Ala molecules (d-Ala′ and d-Ala″) (*upper panel*) and the mDAP (*bottom panel*) modeled on the basis of d-Ala′ and Wat1 are shown in *stick models*. The omit *mF_o_* − *DF_c_* maps for d-Ala′ and d-Ala″ (contoured at 2.0σ) and the side chain oxygen atom of Cys-176 oxidized as the sulfenic acid (contoured at 2.5σ) are colored in *blue*. The oxyanion hole is *asterisked* and also shown as *blue dotted lines* in the *bottom panel. Red dotted lines* indicate the interactions among residues of the catalytic triad. *B*, l,d-carboxypeptidation activities of the wild-type Csd6 (with the treatment of EDTA or not) and the mutants (E110A, Y132A, Y133A, H155A, W158A, H160A, and C176A) with the muramyl tetrapeptide. *C*, SPR experiments with immobilized Csd6 and the muramyl tetrapeptide (or muramyl tripeptide) as an analyte at different concentrations (15.6, 31.3, 62.5, 125, 250, and 500 μm) are shown in traces colored as *orange, light blue, purple, green, red,* and *blue*, respectively.

By analogy with the first carboxypeptidation step of Ldt_Mt2_, His-160 of Csd6 serves as a proton acceptor to assist a nucleophilic attack by Cys-176. In the present Csd6 structure, the side chains of His-160 and Cys-176 are suitably predisposed for a cooperative action with the main-chain carbonyl of Gly-161 (Fig. 5*A*). His-160^Nϵ2^ interacts with Cys-176^Sγ^ with a distance of 3.96 Å and His-160^Nδ1^ interacts with the main-chain oxygen atom of Gly-161 with a distance of 2.75 Å (Fig. 5*A*). In addition, the l,d-CPase domain of Csd6 shows a well defined oxyanion hole to facilitate catalysis. The backbone amide nitrogen atoms of Lys-174, Gly-175, and Cys-176 (*asterisks* in Fig. 5*A*) could stabilize the transient oxyanion species of the polarized carbonyl bond, as in Ldt_Mt2_ (70).

To understand the structural determinants for ligand binding, the structure of Csd6 bound with the reaction product d-Ala (Csd6-Ala in Table 1) was determined. In the Csd6-Ala model, two d-Ala molecules are well ensconced within the active site of monomer A with clear electron density (marked as d-Ala′ and d-Ala″ in Fig. 5*A*), whereas only one d-Ala molecule is present in monomer B at the corresponding position as d-Ala′ in monomer A. The different binding states of monomers A and B in the crystal seem to be correlated with the oxidation state of Cys-176. Its side chain is oxidized to sulfenic acid only in monomer A, as indicated by the extra electron density (*C176*_OH_ in Fig. 5*A*). An oxygen atom of sulfenic acid possibly stabilizes the weak binding of the product d-Ala″^N^ in monomer A. d-Ala′ is located between loops I and III, whereas d-Ala″ is trapped by loops II–IV, right in front of the nucleophile Cys-176. d-Ala′ is too far from the predicted site of the scissile bond for Cys-176 to act, but it interacts with Lys-107^Nζ^, Glu-110^Oϵ2^, His-155^N,O,Nδ1^, and Gly-111^N^, as well as Lys-174^N^, and the main-chain oxygen atom of Leu-113 via a water molecule. Interestingly, the side-chain portion of *m*DAP, part of the Csd6 substrate muramyl-l-Ala^1^-γ-d-Glu^2^-*m*DAP^3^-d-Ala^4^, is structurally identical to a d-Ala molecule. Therefore, we suggest that d-Ala′ and d-Ala″ represent the portion of the substrate and the end product, d-Ala, respectively, for the link between *m*DAP^3^ and d-Ala^4^ (the scissile peptide bond) of the muramyl tetrapeptide. *m*DAP was modeled into the Csd6 l,d-CPase domain on the basis of the positions of d-Ala′ and Wat1 bound in the oxyanion hole. The modeled *m*DAP fits well into the active-site cleft (*bottom panel* of Fig. 5*A*). It makes proper interactions with the oxyanion hole and is suitably positioned for a nucleophilic attack by Cys-176, thus providing the substrate-binding mode. Dissociation constants (*K_D_*) of Csd6 for its complex with d-Ala (or *m*DAP) have also been measured to be 224 (or 94) μm using SPR measurements. The measured association and dissociation rate constants (*k_a_* and *k_d_*) are 1.05 m^−1^ s^−1^ and 2.30 × 10^−4^ s^−1^ for d-Ala and 1.49 m^−1^ s^−1^ and 1.40 × 10^−4^ s^−1^ for *m*DAP, respectively.

To further confirm the role of potential key residues in the active-site cleft, seven single amino acid mutant proteins (E110A, Y132A, Y133A, H155A, W158A, H160A, and C176A) were prepared. When the l,d-carboxypeptidation activities of the wild-type Csd6 and the mutants against the muramyl tetrapeptide for a limited time (30 min) were compared, mutation of Glu-110, Tyr-133, His-155, and Trp-158, as well as His-160 and Cys-176 that belong to the catalytic triad, resulted in a complete loss of the l,d-carboxypeptidation activity (Fig. 5*B*). Glu-110, Tyr-133, and Trp-158 are strictly conserved in *C. jejuni* Pgp2 as well (Fig. 4*E*). Lack of l,d-carboxypeptidation activity in E110A and H155A mutants supports that Glu-110 and His-155 recognize the side chain of *m*DAP, as shown in d-Ala′ bound to Csd6 and *m*DAP modeled into the position of d-Ala′ (Fig. 5*A*). Trp-158 holds the aliphatic side chain of d-Ala″, whereas Tyr-133 likely interacts with the main chain of *m*DAP, possibly via a water molecule such as Wat2 in Fig. 5*A*. Meanwhile, the retained activity of the Y132A mutant indicates that Tyr-132 is not important for the l,d-carboxypeptidation activity of Csd6 (Fig. 5*B*). The l,d-carboxypeptidation activity of Csd6 is independent of metal ions, as there is no change upon EDTA treatment (Fig. 5*B*).

To estimate the kinetics and affinity of Csd6 with the muramyl tetrapeptide (as a substrate) and muramyl tripeptide (as a product), SPR experiments were conducted. The association rate constant (*k_a_*) and the dissociation rate constant (*k_d_*) for the muramyl tripeptide are roughly 3 and 50× larger than those for the muramyl tetrapeptide, respectively (Fig. 5*C*). As a consequence, the *K_D_* values for the muramyl tetrapeptide and the muramyl tripeptide are 27.0 and 510 μm, respectively, indicating that the muramyl tripeptide dissociates readily from Csd6 as the product of Csd6-catalyzed l,d-CPase reaction. This result is also consistent with the muramyl tripeptide not being used as the substrate for the possible l,d-TPase cross-linking reaction in the subsequent step.

##### C-terminal NTF2-like Domain Has a Putative Binding Pocket for Pseudaminic Acid

**FIGURE 6.**
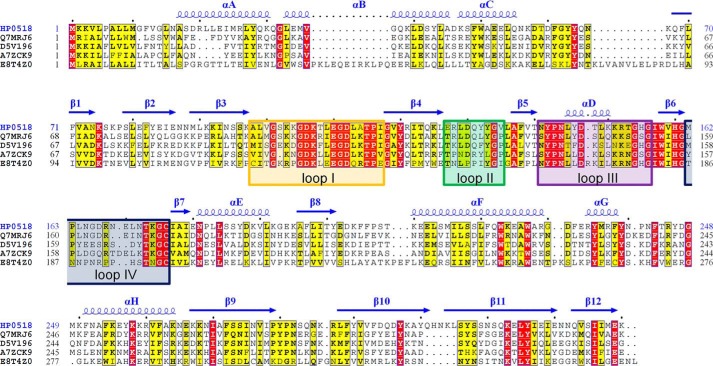
**Sequence alignment of Csd6 homologs.** Sequence alignment of Csd6 homologs from *H. pylori* strain 26695 (HP0518; Swiss-Prot accession code O25255), from *Wolinella succinogenes* (Q7MRJ6), from *Arcobacter nitrofigilis* (D5V196), from *Campylobacter concisus* strain 13826 (A7ZCK9), and from *Thermovibrio ammonificans* (E8T4Z0) was performed and presented by ClustalX (78) and ESPript (79). The secondary structure of Csd6 is presented *above* the aligned sequences. The four loops I–IV around the active site of the Csd6 l,d-CPase domain are indicated with *boxes* colored as in Fig. 4*E*.

**FIGURE 7.**
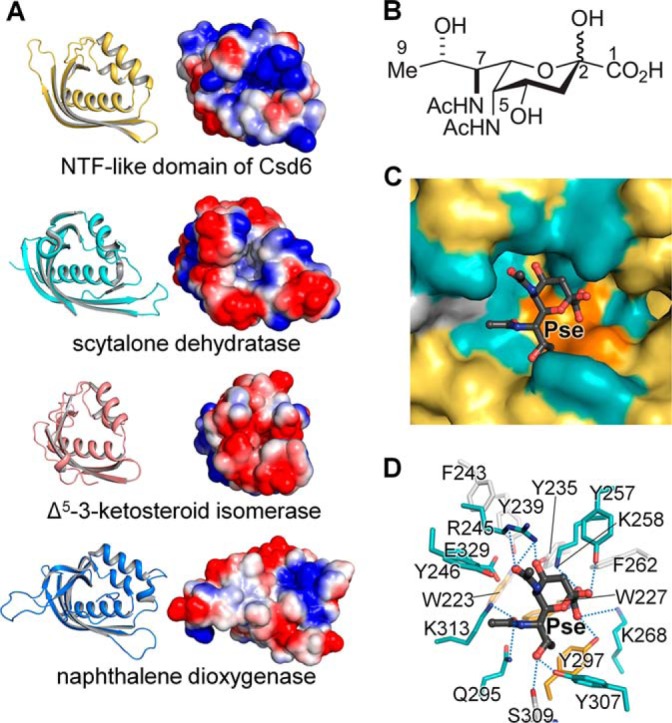
**NTF-like domain of Csd6.** *A*, ribbon diagrams and electrostatic potential surface diagrams of the NTF-like domains of Csd6, scytalone dehydratase, Δ^5^-3-ketosteroid isomerase, and naphthalene dioxygenase. *B*, chemical structure of Pse. *C* and *D*, *in silico* docking of Pse onto the pocket in the NTF-like domain of Csd6. The potential binding pocket is shown in surface representation (*C*), and the residues around the docked ligand are shown as *stick* models (*D*).

*H. pylori* Csd6 is distinct from the known l,d-TPases by having a C-terminal NTF2-like domain (residues Thr-209–Lys-330), following its l,d-CPase domain. The combination of these two domains is conserved in the so-called ErfK/YbiS/YcfS/YnhG family proteins from both *Helicobacter* and *Campylobacter* species (Fig. 6). It was reported that *csd6* gene-deficient transposon mutants of *H. pylori* G27 exhibited altered motility in comparison with its parental strain, and the altered pattern of motility resulted from elevated levels of *O*-linked FlaA Pse-glycosylation of flagellin (40). *H. pylori* flagellin filaments are post-translationally modified by glycosylation with Pse, a nine carbon sugar derivative that resembles sialic acid, which is typically found on mammalian cell surfaces (Fig. 7*B*) (37, 38). The transposon insertions causing the altered motility were located at either positions +765 or +803 bp from the 5′ terminus of the *csd6* gene (40), corresponding to the region encoding the NTF2-like domain. Furthermore, the recombinant *H. pylori* G27 Csd6 protein expressed in *E. coli* was capable of deglycosylating the purified FlaA protein (40). These results suggest a possible function of the Csd6 NTF2-like domain as a deglycosylase, *i.e.* pseudaminidase.

The present structure shows that the overall fold of the Csd6 NTF2-like domain differs from other nonviral sialidase or pseudaminidase of the six-bladed β-propeller fold (52, 71). Instead, it is shared with other NTF2-like superfamily members such as the *Magnaporthe grisea* scytalone dehydratase (72) (PDB code 1IDP; a *Z*-score of 14.3 and a sequence identity of 8% for 118 eq Cα positions), *Pseudomonas putida* Δ^5^-3-ketosteroid isomerase (73) (PDB code 1OPY; a *Z*-score of 12.0 and a sequence identity of 8% for 103 eq Cα positions), and *Rhodococcus* sp. naphthalene dioxygenase (74) (PDB code 2B1X; a *Z*-score of 14.1 and a sequence identity of 7% for 116 eq Cα positions) (Fig. 7*A*). None of these enzymes bears any significant sequence or functional homology to each other, except that they share common hydrophobic substrates. This suggests that the fold of NTF2-like proteins may be a suitable scaffold for building a pocket to bind hydrophobic ligands. Although the Csd6 NTF2-like domain differs considerably from these enzymes in the sequence and the potential function, it shares a deep, largely hydrophobic pocket that is open to the solvent (Fig. 7, *A* and *C*). This potential ligand-binding pocket is lined with numerous hydrophobic residues (**Trp-223**, **Trp-227**, **Tyr-235**, **Tyr-239**, **Phe-243**, Tyr-246, Tyr-257, **Tyr-297**, and Tyr-307). In addition, four positively charged residues (Arg-245, **Lys-258**, Lys-268, and **Lys-313**), and a negatively charged residue (**Glu-329**) are located within the pocket (Fig. 7*D*). The bold-face residues are strictly conserved in Csd6 homologs in ϵ-proteobacteria (Fig. 6). The pocket is somewhat elongated in its shape, unlike hemispherical pockets in scytalone dehydratase and Δ^5^-3-ketosteroid isomerase (Fig. 7, *A* and *C*). The Csd6 NTF2-like domain is highly basic with a calculated pI of 9.4; the electrostatic potential surface shows a highly positively charged surface around its deep pocket (Fig. 7*A*), compared with the above three NTF2-like superfamily enzymes.

In light of the previous report that *H. pylori* Csd6 is involved in the deglycosylation of flagellin (40) and the presence of a deep pocket in the NTF2-like domain of Csd6 for possible binding of a hydrophobic ligand with an acidic group(s) such as Pse, an *in silico* docking study has been performed. It shows that Pse can fit well into the pocket of the NTF-like domain (Fig. 7*C*). However, it remains to be determined whether Csd6 has pseudaminidase activity.

## Discussion

We have determined the first crystal structure of Csd6, one of the cell shape-determining proteins in *H. pylori*. Each of its three domains is associated with a distinct function. The NTD plays a dominant role in homodimerization. The C-terminal NTF2-like domain has a putative binding pocket for Pse. The central l,d-CPase domain has the l,d-TPase fold but the shape of its active-site pocket explains why Csd6 possesses only the l,d-carboxypeptidation activity, despite the presence of the conserved catalytic residues for the potential transpeptidase reaction.

*H. pylori* Csd6, as well as its close homolog Pgp2 from *C. jejuni*, contributes to the helical morphology of the cells. Interestingly, *H. pylori* Csd6 and *C. jejuni* Pgp2 are unrelated in their sequence and structure to previously characterized l,d-CPases such as LdcA and LdcB from other microorganisms. They are thus presumed to form a third family of l,d-CPases. However, it is currently impossible to assign and distinguish genuine l,d-TPases from the functional l,d-CPases via bioinformatics analyses of amino acid sequences alone. Our finding that Csd6-specific active-site loops I and III are much longer than the corresponding loops in known l,d-TPases (Fig. 4*E*) enables us to distinguish l,d-CPases possessing the l,d-TPase fold from *bona fide* l,d-TPases. Indeed, when amino acid sequences of Csd6 homologs in ϵ-proteobacteria are aligned, Csd6-specific loops I and III display high levels of sequence conservation (Fig. 6). In light of this observation, the current annotation of *H. pylori* Csd6, as well as its homologs in ϵ-proteobacteria, as belonging to the protein family containing an l,d-TPase catalytic domain needs to be revised. We propose that they should be classified as a third distinct family of l,d-CPase, as initially noted by others (75).

Enhanced cell attachment of *H. pylori* leads to an increased immune response of the host (76). Too much attachment may thus be detrimental to the bacterium, suggesting a role for motility in the proper balance of bacterial levels during infection. This is intriguing, given that *H. pylori* Csd6 is known to be involved in regulating motility through trimming of the peptidoglycan-derived peptides as an l,d-CPase or through affecting the Pse glycosylation level of flagellin. This study shows that the active-site of the Csd6 l,d-TPase domain is tailored to function as the l,d-CPase, which converts muramyl tetrapeptides into muramyl tripeptides. As Csd6 is nonfunctional as l,d-TPase, muramyl tripeptides in the peptidoglycan cannot be 3→3 cross-linked. This is consistent with the previous reports that the *Helicobacter* peptidoglycan layer lacks 3→3 cross-links (15, 20, 33). An *in silico* docking shows that the Csd6 NTF-like domain presents a potential binding site for Pse, supporting its role in the control of the glycosylation level of flagellin. Relaxation of peptidoglycan 4→3 cross-linking was reported to promote *H. pylori*'s helical shape and stomach colonization (20). Csd6 plays a critical role in determining the helical cell shape of *H. pylori*, because it can limit the supply of substrates for peptidoglycan 4→3 cross-linking. In conclusion, our study on the multidomain, multifunctional Csd6 provides further insights into the strategy of *H. pylori* for regulating its helical cell shape and motility, which are crucial for its virulence.

## Author Contributions

H. S. K., H. N. I., S. M., S. C., N. K. L., S. J. K., J. Y. K., B. W. H., B. I. L., and S. W. S. designed the study and wrote the paper. H. N. I., D. R. A., J. Y. Y., J. Y. J., and H. J. Y. purified and crystallized Csd6 protein and determined its x-ray structure. S. M., D. H., and M. L. synthesized the muramyl peptides. J. Y., M. C., and S. C. conducted docking calculations. C. K. and N. K. L. performed the ALEX-FRET experiment. S. J. K. performed the equilibrium sedimentation experiment. J. Y. K. and G. B. performed mass spectrometric assays of enzyme activities. All authors analyzed the results and approved the final version of the manuscript.

## Footnotes

5 The abbreviations used are:

*m*DAP: *meso*-2,6-diaminopimelate
d,l-CPase: d,l-carboxypeptidase
d,d-CPase: d,d-carboxypeptidase
l,d-TPases: l,d-transpeptidases
l,d-CPase: l,d-carboxypeptidase
Pse: pseudaminic acid
NTD: N-terminal domain
NTF2: nuclear transport factor 2
EMTS: ethyl mercury thiosalicylate
IFD: induced fit docking
Neu5Ac2en: 2-deoxy-2,3-dideoxy-*N*-acetylneuraminic acid
r.m.s.: root-mean-square
MALDI-QIT-TOF MS: matrix-assisted laser desorption/ionization quadrupole ion trap time-of-flight mass spectrometer
SPR: surface plasmon resonance
ASU: asymmetric unit
ALEX: alternating-laser excitation
PDB: Protein Data Bank.
